# Electrochemical aptasensor for the selective detection of vancomycin based on nanostructured “in-lab” printed electrodes

**DOI:** 10.1007/s00604-025-06952-1

**Published:** 2025-01-25

**Authors:** Malek Bibani, Magdolna Casian, Bogdan Feier, Diana Bogdan, Oana Hosu-Stancioiu, Nadia Ktari, Rafik Kalfat, Cecilia Cristea

**Affiliations:** 1 Department of Analytical Chemistry, Faculty of Pharmacy, “Iuliu Hațieganu” University of Medicine and Pharmacy, 4 Pasteur Street, 400349 Cluj-Napoca, Romania; 2 Laboratoire Matériaux, Traitement Et Analyse, INRAP, BiotechPole Sidi-Thabet, 2020 Ariana, Tunisia; 3 National Institute for Research and Development of Isotopic and Molecular Technologies, 67-103 Donat St., 400293 Cluj-Napoca, Romania

**Keywords:** Vancomycin detection, Electrochemical aptasensor, EIS, Gold nanostructure, Cauliflower-shaped gold nanostructures, Antibiotic resistance

## Abstract

A label-free, flexible, and disposable aptasensor was designed for the rapid on-site detection of vancomycin (VAN) levels. The electrochemical sensor was based on lab-printed carbon electrodes (C-PE) enriched with cauliflower-shaped gold nanostructures (AuNSs), on which VAN-specific aptamers were immobilized as biorecognition elements and short-chain thiols as blocking agents. The AuNSs, characterized by scanning electron microscopy (SEM) and atomic force microscopy (AFM), enhanced the electrochemical properties of the platform and the aptamer immobilization active sites. The developed disposable aptasensor allowed label-free detection of VAN via electrochemical impedance spectroscopy (EIS) across a wide range of concentrations (50–1000 nM), with a limit of detection (LOD) of 1.721 nM. The aptasensor presented good selectivity against some commonly found interferences in human serum and milk and was successfully applied to the analysis of these samples.

**Graphical Abstract:**

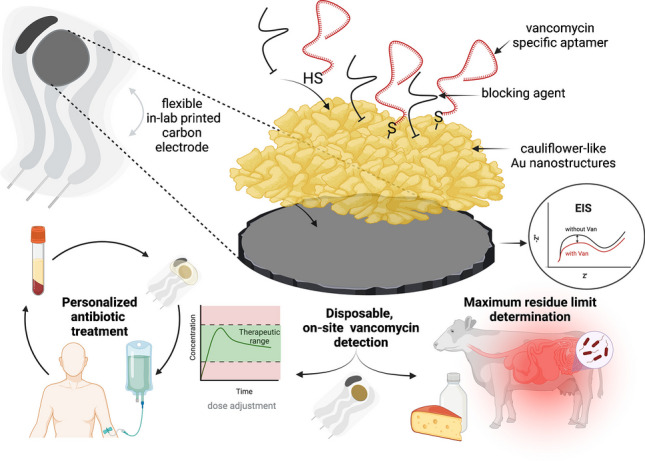

**Supplementary Information:**

The online version contains supplementary material available at 10.1007/s00604-025-06952-1.

## Introduction

Glycopeptide antibiotics have been a key weapon in the fight against bacterial infections for over half a century and are the most frequently used antibiotics for the treatment of severe infections, such as endocarditis, pneumonia, and meningitis [1, 2]. Vancomycin (VAN) is an important antibiotic, used as a first-line treatment for infections caused by antibiotic-resistant Gram-positive pathogens, particularly methicillin-resistant *Staphylococcus aureus* (MRSA) and *Enterococcus sp.* [3]. As one of the “emergency” antibiotics, VAN is used in both human and veterinary medicine to treat severe infections, administered intravenously through infusion and orally for local effect. It is characterized by a narrow therapeutic index and is associated with a relatively high occurrence of nephrotoxicity and ototoxicity. If used at sub-therapeutic concentrations, VAN helps the spread of antimicrobial resistance (AMR). Therefore, therapeutic drug monitoring (TDM) for VAN is recommended [4]. Recently, a correlation was observed between VAN sweat concentration and serum concentration [5], thus, a noninvasive TDM could be achieved using wearable sensors.

Although the use of VAN in animals is limited in order to impair the spread of AMR [6], its excellent antibacterial activity encourages the use of VAN as a feed additive or veterinary drug to treat bacterial infections, especially cow mastitis. VAN abuse may lead to potential residues in milk or in the environment, thus favoring the AMR [7]. To combat this concern and ensure that milk entering the food supply adheres to regulatory standards, the European Union has set maximum residue limits (MRLs) for antibiotics that are still allowed to be administered to animals, along with the additional requirement of implementing a withdrawal period of antibiotics in animal food products to avoid food safety problems [8].

Traditional antibiotic detection techniques include methods based on chromatography coupled with mass spectrometry [9, 10], microbiological assays [11, 12], and enzyme-linked immunosorbent assays [13, 14], which are expensive, time-consuming, and involve laborious pretreatment protocols, long assay time, and lack of portability. Although ELISA kits have been made available for easier public use, there are still concerns that need to be addressed, including enzyme inactivation, cross-reactivity, and long detection times, which are unsuitable for antibiotic monitoring in real-world situations [15].

In the area of animal health management, biosensors have come into the spotlight as versatile and innovative devices, gaining recognition in the global market. Currently, precision livestock farming techniques are applied in sweat and salivary sensing, serodiagnosis, and animal product safety monitoring [16].

Electrochemical sensors offer distinct advantages over conventional methods for the detection of antibiotics, making them a superior choice for certain applications [17]. One key advantage is their remarkable sensitivity, which enables the detection of analytes at low concentrations. The real-time nature of the electrochemical measurements allows rapid and dynamic monitoring, facilitating timely responses in situations where rapid analysis is crucial. In addition, electrochemical sensors often exhibit excellent selectivity, minimizing the interferences from complex samples. Their relative simplicity, portability, wearability, flexibility, and cost-effectiveness make them particularly attractive for on-site and point-of-care applications, allowing the analysis of different types of samples (human serum, sweat, food, and environmental samples). Furthermore, electrochemical sensors can be easily miniaturized (allowing their integration into various automated devices for continuous monitoring) and modified with nanomaterials and (bio)recognition elements (leading to great sensitivity and selectivity). Various gold nanostructures (AuNSs) can be (electro)chemically synthesized, enhancing the catalytic and electrical properties, the active surface for aptamer immobilization through Au–S bonds and the interaction of the aptamer with the target. Overall, all these attributes make electrochemical sensors advantageous tools for efficient and reliable antibiotic detection in diverse settings [18, 19].

Aptamers, often referred to as “chemical antibodies,” are short, single-stranded DNA or RNA sequences that exhibit high affinity and specificity for a specific target molecule. In electrochemical sensors, aptamers play a crucial role because of their unique advantages. One key advantage is their ability to recognize specific targets, including small molecules, proteins, and whole cells, making them versatile for various sensing applications [20, 21]. Aptamers offer a cost-effective and more stable alternative to traditional antibodies, with the added benefit of easier synthesis and functionalization, with few batch-to-batch variations. Their stable and well-defined three-dimensional structures and target-induced conformational changes contribute to their enhanced sensitivity and selectivity in electrochemical sensing, making them ideal candidates for use as biorecognition elements [22]. Moreover, aptamers can be easily immobilized on electrode surfaces, thereby providing a stable and reproducible sensing platform. This immobilization process facilitates the development of robust and reusable electrochemical sensors, contributing to the cost-effectiveness and sustainability of sensing technologies [23]. Overall, the unique properties of aptamers make them valuable components for the design and development of highly efficient electrochemical sensors for a wide array of applications in fields such as medical diagnosis, environmental monitoring, and food safety.

This study developed an innovative, sensitive, and disposable aptasensor for on-site VAN detection. The electrochemical sensor was based on an in-lab printed carbon electrode (C-PE) functionalized with gold nanostructures (AuNSs), on which a VAN-specific aptamer was grafted as a biorecognition element. Building on the stability and reproducibility of aptamers, the in-lab printed carbon electrodes (C-PEs) represent an important aspect of this study, significantly enhancing the versatility and cost-effectiveness of the sensing platform. C-PEs reduce dependence on expensive commercial options and provide greater accessibility and customization, allowing researchers to design and tailor them to specific experimental needs with precise control over dimensions, materials, and configurations. The flexible support for C-PEs further facilitates their use in wearable sensors, offering a convenient and affordable starting point. Thus, in-lab C-PEs align with the growing emphasis on sustainable and resource-efficient methodologies.

The AuNSs electrosynthesis was optimized and the AuNSs were characterized by scanning electron microscopy (SEM) and atomic force microscopy (AFM). The aptamer-VAN affinity was determined using isothermal titration calorimetry (ITC) and surface plasmon resonance (SPR). Each step in the aptasensor fabrication and optimization process was characterized electrochemically by cyclic voltammetry (CV), differential pulse voltammetry (DPV), and electrochemical impedance spectroscopy (EIS).

Based on the fact that the binding of VAN to the aptamer induces changes at the electrode surface that alter the electron transfer of the ferro/ferricyanide redox probe, which is then measured using EIS, the quantification of VAN was assessed using the EIS spectra in the presence of 5 mM ferro/ferricyanide as redox probe, obtaining an aptasensor with LOD in the nanomolar range. The developed aptasensor presented good selectivity against some commonly found interferences in serum and milk and was successfully applied for the analysis of these samples. To the best of our knowledge, this is the first electrochemical sensor that combines the advantages of in-lab C-PEs, AuNSs, and aptamers for VAN detection from milk and human serum.

VAN is currently the antibiotic of choice in severe cases of cow mastitis and the occurrence of antibiotic residues in animal products can produce significant health hazards to consumers, together with portable potentiostats, this platform could be a great starting point for the fabrication of easy-to-use, disposable, on-site rapid testing devices for the assessment of possible contaminations that could occur in milk and derivates.

For therapeutic drug monitoring, given the therapeutic range of this antibiotic (10–20 mg/L or ~ 7–14 µM), our sensor matches the criteria required for safety and efficacy assessment. Moreover, given the recent advances in personalized medicine using wearable noninvasive sensors, along with the correlation found between VAN concentrations in plasma and sweat [16], the flexible design of our sensor could be a starting point for the development of a noninvasive wearable next-generation point-of-care monitoring system that could provide sweat-based real-time VAN treatment monitoring.

## Materials and methods

### Materials

All chemicals were of analytical grade and used without further purification. Sulfuric acid (H_2_SO_4_), hydrochloric acid (HCl), sodium hydroxide (NaOH), sodium chloride (NaCl), magnesium chloride hexahydrate (MgCl_2_**·**6H_2_O), potassium chloride (KCl), sodium carbonate (Na_2_CO_3_), sodium hydrogen phosphate dodecahydrate (Na_2_HPO_4_**·**12H_2_O), sodium dihydrogen phosphate monohydrate (NaH_2_PO_4_**·**H_2_O), tetrachloroauric (III) acid (HAuCl_4_), tris(hydroxymethyl)aminomethane (TRIS), tris(2-carboxyethyl)phosphine hydrochloride (TCEP), potassium hexacyanoferrate (II) trihydrate K_4_[Fe(CN)_6_]·3H_2_O), potassium hexacyanoferrate (III) (K_3_[Fe(CN)_6_]), 6-mercapto-1-hexanol (MCH), histidine, lactose (Lac), glucose (GLU), and human serum were purchased from Sigma-Aldrich (Germany).

Vancomycin hydrochloride (VAN) was obtained from Linaris Biologische Produkte GmB (Frankenweg, Germany), and gentamicin sulfate (Gen) from Bioworld (Louis Park, MN, USA). The three different types of milk used in this study (low-fat, 1.5%, and 3% fat) were purchased from a local supermarket.

The VAN-specific aptamer (Apt) used in this study was previously selected [24] and synthesized by Eurogentec (Belgium) with a customized thiol group at the 5′ end and a ferrocene group at the 3′ end, having the following sequence: 5′-SH-(CH_2_)_6_-CGAGGGTACCGCAATAGTACTTATTGTTCGCCTATTGTGGGTCGG-Ferrocene-3′.

The supporting electrolyte solutions used in this study included a 0.1 M KCl solution and a TRIS buffer (pH 7.4) containing 10 mM TRIS, 100 mM NaCl, 100 mM KCl, and 10 mM MgCl_2_·6H_2_O. All solutions were prepared using UltraPure™ DNase/RNase-free distilled water (Thermo Fisher, USA). The lyophilized Apt was resuspended in 10 mM TRIS buffer pH 7.4 to a stock solution of 100 µM, aliquoted, and stored at –20 °C. The HAuCl_4_ and histidine solution mixture was prepared in 0.5 M H_2_SO_4_. For MCH, a 10 mM initial stock was prepared in TRIS buffer containing 20% ethanol, after which further dilutions were performed using TRIS buffer alone. For VAN analysis, fresh concentrations of VAN were prepared in TRIS buffer. For the analysis of real samples, human serum (Sigma-Aldrich) and milk (from a local supermarket) were used without further filtration.

### Methods and instruments

All electrochemical experiments were performed using an Autolab PGSTAT302N potentiostat (Metrohm Autolab, The Netherlands) equipped with Nova 1.11 software. The heating of the Apt was performed using an Eppendorf Thermomixer® C. The pH measurements were carried out using a micropH meter (Hanna Instruments).

Isothermal titration calorimetry (ITC) was performed using an Affinity ITC microcalorimeter (TA Instruments, New Castle, USA) controlled by ITCRun v.3.8.4.24000 and Nano&Affinity ITC Data Collection software. For degassing the solutions, the compatible Degassing Station (TA Instruments, New Castle, USA) was used. The thermodynamic parameters were obtained by fitting the titration curves against the built-in independent sites using NanoAnalyze v.3.12.5 software.

The surface plasmon resonance (SPR) experiments were performed on a three-channel Biosensing BI-2500 instrument using the compatible bare gold chips (Biosensing Instrument Inc., Tempe, AZ, USA). To extract the SPR kinetic parameters, the kinetic data were analyzed in the framework of the Langmuir isotherm 1:1 binding model using BI-software version 2.4.4 (Biosensing Instrument Inc., Tempe, AZ, USA), which includes the software Scrubber (BioLogic Software Pty Ltd., Campbell, Australia).

In this study, in-lab printed electrodes with a carbon-based working surface (C-PE) were used. For the electrode printing process, the carbon conductive ink Electrodag 423SS was purchased from Henkel (Dusseldorf, Germany), and Ag/AgCl ink Electrodag PF-410 was purchased from Acheson (Delaware, USA). An autoclave (Memmert GmbH, Schwabach, Germany) was used for the successive and controlled drying of the conductive ink during the printing process.

Morphological and topographical surface characterization of the developed sensor was performed using atomic force microscopy (AFM). The measurements were conducted on a Cypher S microscope (Asylum Research-Oxford Instruments, Santa Barbara, CA) in tapping mode, in air, under ambient conditions, with silicon probes (AC240TS-R3, Olympus, Japan), reflex side aluminum coated, tip radius typical 7 nm, with a spring constant of 2 N/m (0.6–3.5 N/m) and a resonance frequency of 70(± 20) kHz. Data acquisition and image analysis were performed using the integrated Asylum Research software (AR 16.33.234, Asylum Research) written within the Igor Pro software package (Igor Pro 6.38B01, WaveMetrics, Inc., Lake Oswego, OR, USA). Several areas of the sample surface were analyzed, with 512 pixels/line and with a scan rate of less than 1 Hz. Scanning electron microscopy (SEM) images were obtained using a Hitachi SU8230 SEM (Tokyo, Japan) at 30 kV, 10 µA, and a working distance of 15 mm, using Aztec software from Oxford Analytics.

#### Electrochemical techniques

The electrochemical techniques used in this study were cyclic voltammetry (CV), differential pulse voltammetry (DPV), and electrochemical impedance spectroscopy (EIS). CV (the potential was cycled twice between – 0.2 V and + 1.2 V, with a scan rate of 100 mV/s), DPV (the potential was scanned from – 0.2 and + 0.5 V, with a modulation amplitude of 50 mV, modulation time of 40 ms and a scan rate of 10 mV/s), and EIS (the frequency range was between 0.01 and 100,000 Hz, with an amplitude of 0.01 V and DC potential determined by Open Circuit Potentials) measurements were performed in the presence of 5 mM [Fe(CN)_6_]^3–/4–^ redox probe prepared in 0.1 M KCl solution. The measurements using ferrocene-labeled aptamer were performed in a 0.1 M KCl solution.

#### Aptamer affinity evaluation

##### Isothermal titration calorimetry (ITC)

The ITC analyses were conducted according to the following protocol. Prior to each measurement, the Apt solution was subjected to thermal treatment and each solution was degassed for 10 min under vacuum (25 inHg) at 25 °C using the Degassing Station. For the Apt solution, 300 µL of 10 µM Apt in TRIS buffer was prepared, loaded into the ITC sample cell, and titrated with 150 µM VAN solution prepared in the same buffer. The reference cell of the calorimeter was filled with 300 µL nuclease-free water. For titration, 23 titration steps were performed for each injection using 5 µL of VAN solution, except for the first injection, for which 2 µL was used. The other titration parameters were as follows: initial baseline (300 s), injection interval (150 s), stirring rate (100 rpm), and temperature setpoint (25 °C).

##### Surface plasmon resonance (SPR)

For SPR analysis, the bare Au chip was initially washed with ethanol, dried under a nitrogen stream, and hydrated with 300 µL TRIS buffer for 1 h at room temperature. The Au chip was then covered with 300 µL of 1 µM Apt solution prepared in TRIS buffer (previously reduced and thermally activated) and left overnight at 4 °C in a water-saturated atmosphere. Subsequently, the surface was rinsed with 300 µL of TRIS buffer and incubated again for 30 min at room temperature with 300 µL of 100 µM MCH solution prepared in the same buffer. After a final wash with 300 µL of TRIS buffer, the modified Au chip was mounted on the SPR prism.

The running buffer for the affinity evaluation was TRIS buffer, maintaining a constant flow rate of 60 µL/min. Prior to analysis, the buffer was filtered through a 0.2 µm pore diameter filter and degassed for 1 h under vacuum (25 inHg) at 25 °C using the Degassing Station. After achieving a stable baseline, increasing concentrations (0.05–10 µM) of VAN solutions prepared in TRIS buffer were injected. For each concentration, 300 µL of the solution was injected for 300 s (association phase), followed by 300 s of buffer running through the system before a new injection (dissociation phase and new baseline stabilization). Before the first injection of VAN, 300 µL of TRIS buffer was injected for blank correction.

#### In-lab electrode printing process

The components of the electrochemical cell were printed on a thin flexible polymeric substrate by optimizing the printing procedure described in two previous studies [25, 26]. Briefly, the stencil was placed on top of the polymeric substrate, a layer of conductive ink was applied, and a rubber squeegee was dragged along the stencil to distribute the ink uniformly in the aperture. Ag/AgCl ink was printed first to provide the reference electrode (RE), after which the working (WE) and counter (CE) electrodes were printed using carbon conductive ink. After each printing step, the polymeric substrate was autoclaved for 15 min at 50 °C. To minimize shortcuts on the connections, insulator tape was used to cover the interconnections, after which the resulting C-PEs were left to dry out completely at 50 °C overnight. In addition, serpentine connections were printed using Ag/AgCl conductive ink. Finally, silver wires were attached to the serpentine connection to facilitate the connection between C-PE and the potentiostat.

#### C-PE conditioning and gold nanostructured platform fabrication

The first step in the aptasensor development consisted of the activation of C-PEs with 1 M Na_2_CO_3_ solution using an amperometric procedure by applying a constant potential of + 1.2 V (*vs.* Ag/AgCl) for 600 s. After activation, the C-PEs were modified with gold nanostructures (AuNSs) in the optimal conditions: 10 mM HAuCl_4_ solution in 0.5 M H_2_SO_4_ containing 150 mM histidine by chronopotentiometry (CP)-assisted electrodeposition (− 100 µA current applied for 600 s). After modification, the electrode was rinsed three times with 100 µL of nuclease-free water.

#### Aptasensor development

The obtained gold nanostructured platform (C-PE/AuNSs) was further used for the immobilization of the VAN-specific aptamer through a gold-thiol covalent bond. Two different immobilization methods were tested: (i) overnight incubation at 4 °C and (ii) multipulse pulse amperometry (MPA). For MPA, the applied potential was switched between + 0.5 V and –0.2 V (*vs.* Ag/AgCl) with a 10 ms pulse duration for 300 s.

Prior to use, the possible disulfide bonds formed during Apt storage were reduced with TCEP, by incubating at room temperature for 1 h in the dark, a 1:1 (*v/v*) mixture of 100 µM Apt solution with 20 mM TCEP, both prepared in TRIS buffer. The reduced aptamer was then diluted with TRIS buffer to a final concentration of 1 µM and subjected to thermal treatment by heating to 95 °C for 5 min, followed by cooling to –20 °C for 5 min. After Apt immobilization, the electrode was rinsed three times with 100 µL of TRIS buffer.

To prevent non-specific adsorption on the surface of the electrode, the remaining gold active sites were blocked with 20 µL of 100 µM MCH solution prepared in TRIS buffer by incubation for 30 min at room temperature. After blocking, the electrode was rinsed three times with 100 µL of TRIS buffer. The electrode surface was consistently covered during aptasensor development with a drop of TRIS buffer to prevent dehydration and spatial degeneration of the Apt.

The electrode surface was electrochemically characterized after each modification step by EIS and DPV, using a 5 mM [Fe(CN)_6_]^3−/4−^ solution.

#### Vancomycin quantification procedure

For VAN quantification, the obtained sensing platform (C-PE/AuNSs/Apt/MCH) was incubated with 50 µL of various concentrations of VAN prepared in TRIS buffer for 45 min at room temperature in a water-saturated atmosphere. After incubation, the electrode was rinsed three times with 100 µL of TRIS buffer to eliminate the possibility of nonspecific adsorption. Two different VAN quantification procedures were tested, using the changes before and after VAN incubation in the electrochemical signal of the (i) Apt ferrocene label or (ii) a 5 mM [Fe(CN)_6_]^3−/4−^ solution.

For the first procedure, a 50 µL 0.1 M KCl solution was applied on the electrode and the electrochemical signal of the Apt ferrocene label was recorded. The analytical signal was determined based on the anodic current intensity (*I*) values before and after VAN incubation.

For the second quantification procedure, the analytical signal of the redox probe was determined based on the charge-transfer resistance (*R*_ct_) and anodic current intensity (*I*) values before and after target incubation. The EIS and DPV signals were calculated as a percentage of reduction in *R*_ct_, respectively increase in *I* after incubation with VAN, according to the following Eqs. (1) and (2):

1 $${S}_{\text{incubation }}=\frac{\left({R}_{\text{ct MCH}}- {R}_{\text{ct VAN}}\right)\times 100}{\begin{array}{c}{R}_{ct\;MCH}\end{array}}$$

2 $${S}_{\text{incubation}}=\frac{\left|{I}_{\text{ MCH}}- {I}_{\text{ VAN}}\right|\times 100}{{I}_{MCH}}$$

#### Interference studies

For the treatment of serious MRSA infections, VAN is often combined with a second antibiotic, most often gentamicin. In the aptasensor development process, the potential interferents were established depending on the final applicability on real sample analysis (human serum and milk). Therefore, the selectivity of the developed aptasensor in complex matrices was evaluated against gentamicin, glucose, and lactose. The assessment involved examining the EIS signal responses for individual interferent solutions (1.0 µM) and 1:1 mixture with VAN (1.0 µM each), following the protocol described for the VAN quantification procedure (2.3.5).

#### Real sample analysis

To evaluate the performance and applicability of the developed aptasensor for the quantification of VAN in real samples, artificial human serum and milk with different fat contents (0.1%, 1.5%, and 3.5%) were used for analysis. All samples were diluted 10 times with TRIS buffer to ensure the electrolytic composition and ionic strength necessary for proper recognition and binding of the aptamer. The resulting VAN solutions were then incubated for 45 min at room temperature in the same manner as described for VAN quantification. The applicability of the developed sensing method was then evaluated by spiking the diluted samples with a known concentration of VAN (1 µM) and calculating the recovery rates as follows: recovery (%) = C_s_/C_t_ × 100, where C_s_ is the mean VAN concentration of the spiked sample and C_t_ is the expected theoretical concentration of the sample after spiking.

## Results and discussion

### Aptamer affinity studies

**Fig. 1:**
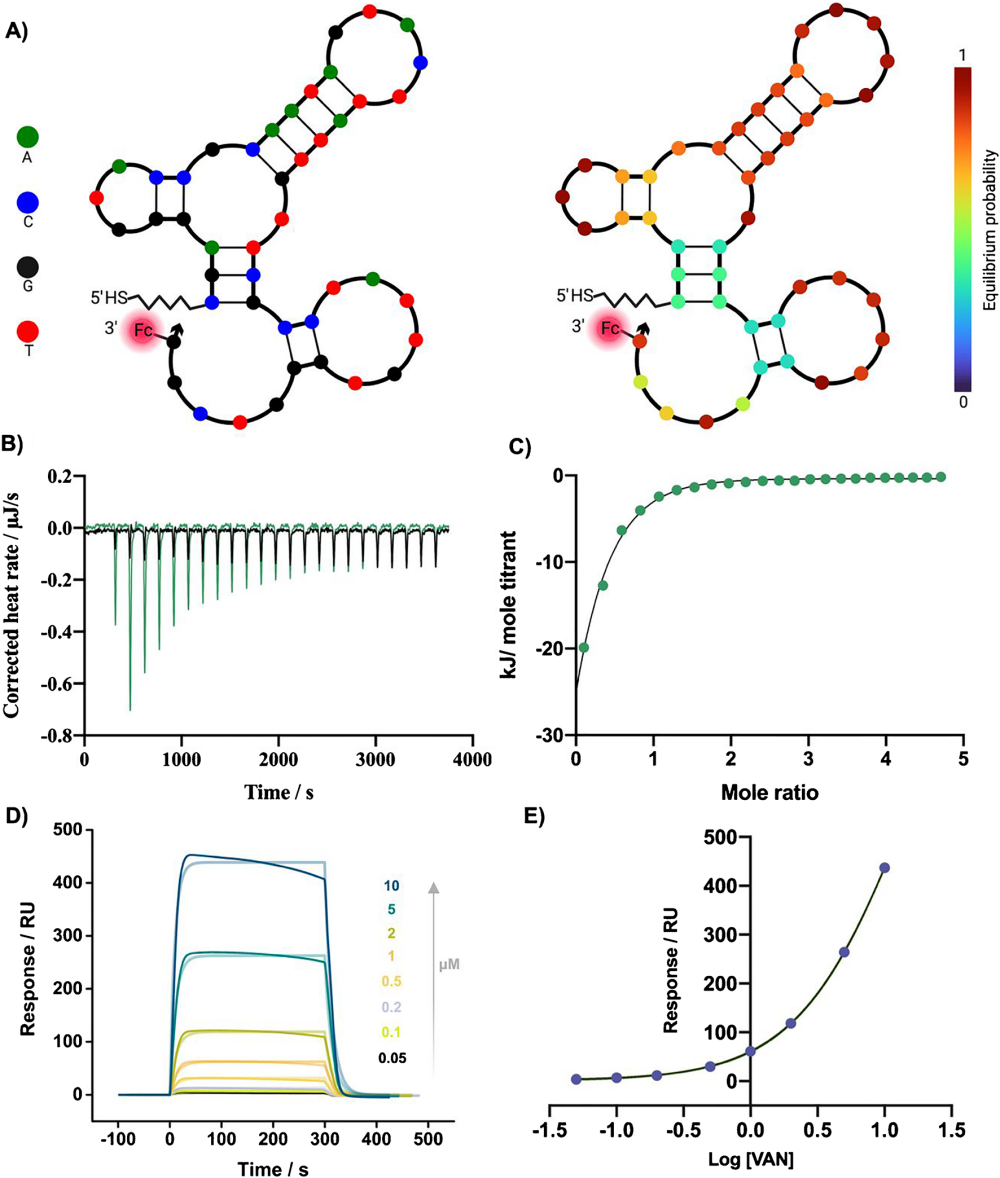
**A** Secondary structure prediction of the modified VAN Apt. The analysis was carried out at 25 °C, with a 0.15 M Na^+^ and 0.01 M Mg^+2^ concentration, using the Nupack web server [28]. **B** ITC measurements: ITC data obtained for the exothermic reaction during titration of 10 µM Apt (green) and 0 µM Apt (black) with 150 µM Van. **C** Integrated heat rates for each injection plotted against the Van Apt mole ratio in the ITC cell; inset: mean thermodynamic and affinity parameter values obtained from three different measurements. **D** SPR analysis: Sensograms representing the kinetic binding profile of VAN-Apt (representative normalized, blank subtracted SPR sensograms for eight concentrations of VAN (0.05, 0.1, 0.2, 0.5, 1, 2, 5, and 10 µM); the colored more transparent lines represent the fit of experimental data to a 1:1 kinetic binding model). **E** Corresponding equilibrium binding curve (fitted to a steady-state affinity model (*n* = 3)). Experimental conditions: 60 µL/min flow rate, injections of 300 s, 100 s dissociation time

Since their discovery in the 90s, many aptamers have been reported in the literature for numerous applications. To be used as a biorecognition element in electrochemical sensing, an aptamer must undergo a binding-induced conformational change that produces a significant change in the electron transfer of the redox probe. The selected VAN-specific aptamer was designed to exhibit this property by destabilizing the parent DNA sequence with the removal of four base pairs from its stem (4trunc), leading to a binding-induced conformational change observed via circular dichroism [27]. The 4trunc aptamer with the most stable predicted secondary conformation, characterized by the lowest Gibbs free energy (ΔG = − 7.03 kcal/mol), is shown in Fig. 1A. Secondary structure prediction was carried out at 25 °C, with a 0.15 M Na^+^ and 0.01 M Mg^2+^ concentration, using the Nupack Web server [28]. The sequence was linked to a thiol functional group via a C_6_ linker, to enable sufficient spacing for aptamer folding and target binding.

When selecting an aptamer from literature, in addition to the characterization presented in the selection paper, it is essential to test whether the aptamer is reliable and works in the designed settings. Therefore, the affinity of the selected Apt sequence, modified with thiol group, was evaluated using two different and complementary affinity evaluation techniques, ITC, and SPR.

ITC is a highly effective technique for studying binding interactions, along with the determination of affinity and thermodynamic constants, in a label-free manner. ITC experiments showed that the binding reaction between the aptamer used in this study and its target molecule was exothermic (Fig. 1B). The affinity and thermodynamic parameters were obtained by further integrating the resulting heat profiles (Fig. 1C): dissociation constant (*K*_D_ = 13.35 ± 2.74 µM), binding enthalpy (ΔH = –40.27 ± 0.73 kJ/mol), and entropy (ΔS = –42.92 ± 1.06 J/mol·K), stoichiometry of the reaction (*n* = 1.10 ± 0.05).

Another technique intensively applied in the study of aptamer binding kinetics is SPR, a surface-sensitive optical technique that measures the refractive index changes due to analyte binding. The SPR experimental setup consisted of the immobilization of the Apt on the surface of a gold chip, blocking off the remaining gold sites with MCH, followed by a series of injections using increasing concentrations of VAN (0.05, 0.1, 0.2, 0.5, 1, 2, 5, 10 µM). The interaction of the immobilized Apt with different concentrations of VAN in solution was monitored, and subsequently processed and analyzed for obtaining both kinetics and equilibrium parameters. The normalized, blank-subtracted sensograms were fit to a 1:1 kinetic binding model (Fig. 1D) and a steady-state affinity model (Fig. 1E). The rate constants obtained were as follows: *k*_on_ 3.44 ± 1.38 × 10^3^ µM^−1^ s^−1^, *k*_off_ 0.064 ± 0.03 × 10^3^ s^−1^. According to the expression *K*_D_ = *k*_off_/*k*_on_, the dissociation constant was calculated to be 18.31 ± 1.15 µM.

The obtained *K*_Ds_ through the two different affinity evaluation methods (ITC and SPR) were similar, as for the *K*_D_ previously reported in the aptamer selection paper (45.5 ± 2.2 µM) [24], the difference can be attributed to the different media used for the affinity evaluation (bloodstream *vs.* TRIS buffer) and the different method applied (calibration-free E-AB sensor *vs.* ITC/SPR).

### Platform design and sensing principle

**Fig. 2:**
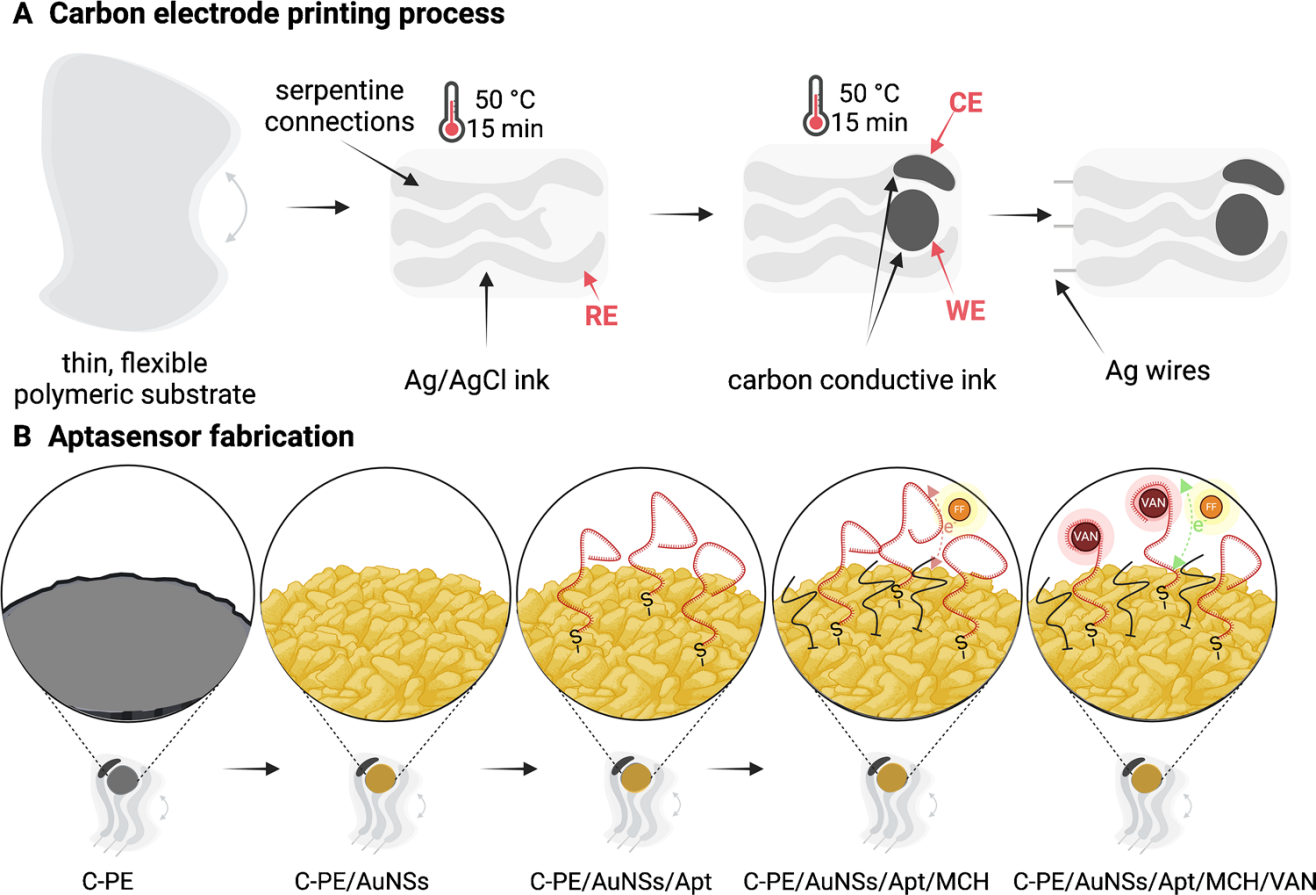
Schematic representation of the aptasensor fabrication process and its working principle

The aptamer-based electrochemical sensing platform used in this study was designed starting from an in-lab-fabricated flexible C-PE functionalized with cauliflower-like AuNSs, on which thiolated VAN-specific Apt was grafted via Au–S covalent bonds. To prevent nonspecific adsorption, the remaining available Au sites were blocked using a short-chain thiol (MCH). The working principle of the developed aptasensor was based on the selection strategy of the chosen aptamer that presented a key stem-loop closing configuration, meaning that the aptamer was selected to specifically respond to the presence of VAN by detaching the complementary part and opening the stem-loop [24, 27]. In the case of our sensing platform, before VAN binding the stem-loop configuration of the aptamer presumably hinders the electron transfer rate, whereas when VAN is present the stem-loop is opened, thus exhibiting a binding-induced change in the electron transfer rate. Moreover, VAN is positively charged at pH 7.4 [29], which could further facilitate the electron transfer rate from the negatively charged redox probe [30]. The schematic illustration of the C-PE printing and aptasensor fabrication process, along with the “signal-on” sensing principle is shown in Fig. 2.

For a preliminary characterization of the obtained C-PEs, a simple CV voltammogram was recorded in 5 mM [Fe(CN)_6_]^3−/4−^ in 0.1 M KCl. As shown in Fig. S1A, the carbon surface did not exhibit clear redox peaks of the [Fe(CN)_6_]^3−/4−^ redox couple; therefore, to enhance and standardize the electrochemical signal, a simple chronoamperometric surface treatment was applied using a 1 M Na_2_CO_3_ solution. After pretreatment, the peak potential separation (*ΔE*_*p*_) changed from 0.64 to 0.15 V, highlighting a lower barrier to electron transfer and electrochemical reversibility.

The size of the in-lab-fabricated C-PEs was 1.5 × 1 cm (length × width), with a geometric surface of the WE of 0.196 cm^2^. The surface area available for electron transfer to the species in solution was calculated using the Randles–Sevcik Eq. (3):

3 $${I}_{p}=\left(2.69\times {10}^{5}\right)\times {n}^{3/2}\times A\times {D}^{1/2}\times {v}^{1/2}\times C$$

where *I*_p_ is the peak current (A), *n* is the number of transferred electrons, *A* is the electroactive area (cm^2^), *D* is the diffusion coefficient of the oxidized species (cm^2^ s^−1^), *v* is the scan rate (V s^−1^), and *C* is the analyte concentration (mol L^−1^). The CV measurements were performed on the bare C-PEs in 5 mM [Fe(CN)_6_]^3−/4−^ in 0.1 M KCl by varying the scan rate from 25 to 300 mV s^–1^ (Fig. S1B). From the slope of the Randles–Sevcik plot (peak currents vs. square root of the scan rate; Fig. S1C), the electroactive area was calculated to be 0.226 cm^2^.

### AuNSs electrodeposition and surface characterization

In the field of biosensors, gold nanoplatforms not only allow easy grafting of specific biorecognition elements via Au–S covalent bonds but also enhance interfacial molecular recognition by accelerating molecular diffusion and reducing steric hindrance [31].

By examining the Au electrodeposition process, two essential phases can be distinguished, nucleation and growth, which are both influenced by the reaction conditions [32]. Thus, optimization of the electrodeposition conditions, such as the electrochemical technique, electrodeposition potential, HAuCl_4_ concentration, and deposition time, influences the regulation of nucleation and directional aggregation. By setting the appropriate conditions, a non-equilibrium system can be created, promoting the formation of AuNSs with distinct architectures.

**Fig. 3:**
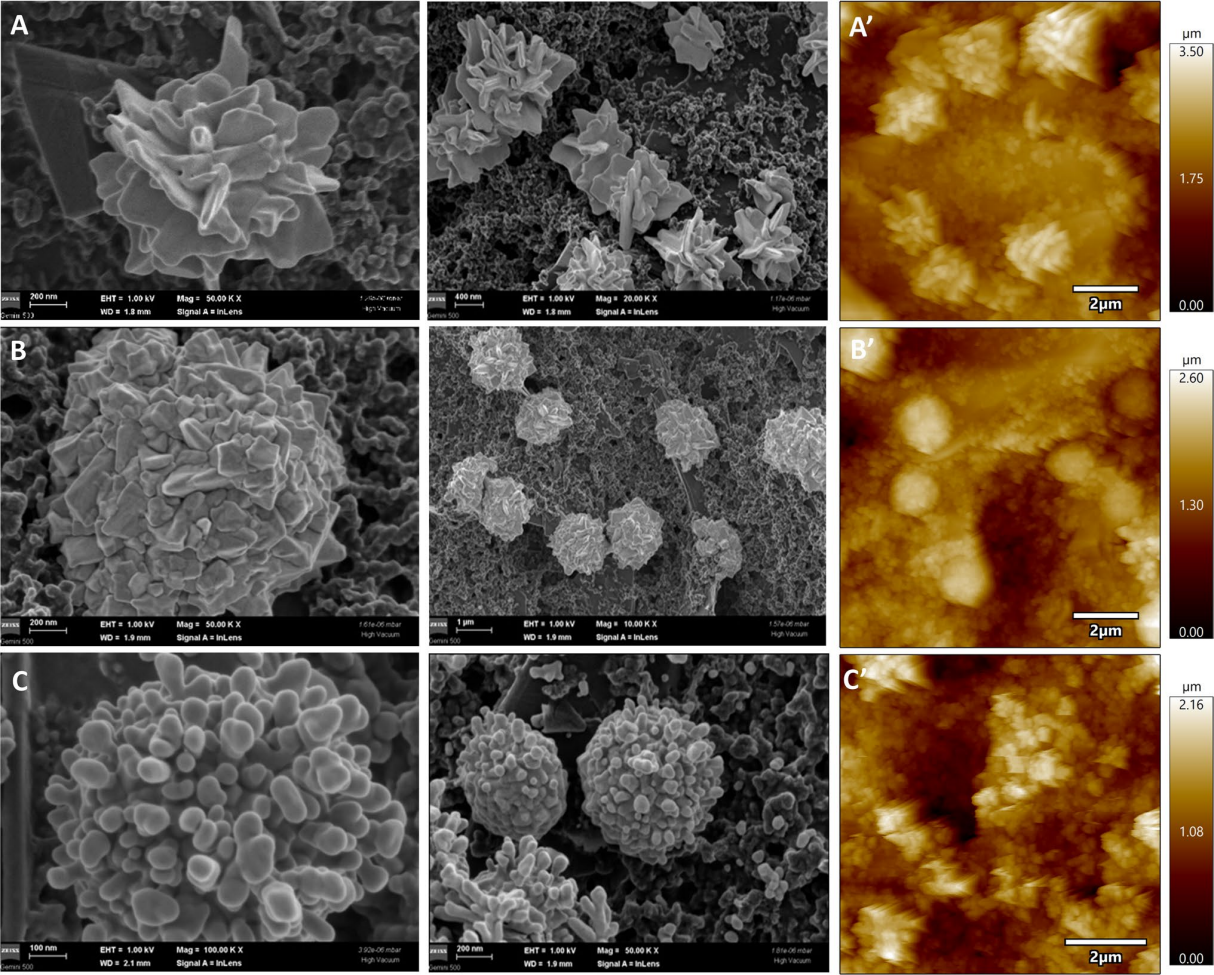
SEM images of the obtained C-PE/AuNSs platforms: thistle-like (obtained using 10 mM HAuCl_4_, 0 mM His, CP –100 µA) (**A**), cauliflower-like (obtained using 10 mM HAuCl_4_, 150 mM His, CP –100 µA) (**B**), and crown-like (obtained using 10 mM HAuCl_4_, 150 mM His, CP –200 µA) (**C**) structures at different magnifications and the corresponding AFM images (**A’**, **B’**, and **C’**, scan size 10 µm, scale bar 2 µm)

Our approach included the optimization of a previously reported AuNSs formation strategy involving the incorporation of amino acids into a HAuCl_4_ solution for controlled Au nanostructure formation on the surface of C-PE. The galvanostatic deposition of AuNSs on screen-printed electrodes by combining HAuCl_4_ with amino acids, such as cysteine [33] and histidine [31], has been previously explored by our group. Those initial conditions served as a starting point for this study and were systematically optimized. Hence, the augmentation of the electrodeposition process for the formation of AuNSs on the sensing surface of C-PE involved the optimization of three key parameters: (i) histidine concentration, (ii) HAuCl_4_ solution concentration, and (iii) electrodeposition time. Under different experimental conditions, AuNSs with various shapes were obtained via one-step electrochemical deposition. Two different negative currents were applied to the electrode (− 100 µA and − 200 µA) in the presence or absence of His to obtain the three-dimensional structures. The morphologies of the resulting AuNSs were characterized by SEM and AFM (Fig. 3).

The first goal of the optimization study was to assess whether the presence of His exerted a crucial influence on the shape or size of the AuNSs. The SEM images (Fig. 3A–C) revealed captivating AuNSs, with distinct morphological differences based on the protocols employed. As can be observed in Fig. 3A, the electrodeposition of Au from a 10 mM HAuCl_4_ at − 100 µA, in the absence of His resulted in the formation of thistle-like structures. Conversely, the addition of 150 mM of His to the electrodeposition solution resulted in the formation of cauliflower-like structures, as shown in Fig. 3B. At more negative current (− 200 µA), crown-like nanostructures formed on the C-PE surface (Fig. 3C).

The AFM images of the obtained three platforms are presented in Fig. 3A’–C’. The surface features identified by AFM corresponded to the surface characteristics observed by SEM. For the thistle-like structures, the height ranged between 0.9 and 1.7 µm, the crown-like structures had a height range of 0.2–1.0 µm, whereas the cauliflower-like nanostructures reached heights of 0.5–1.0 µm.

**Fig. 4:**
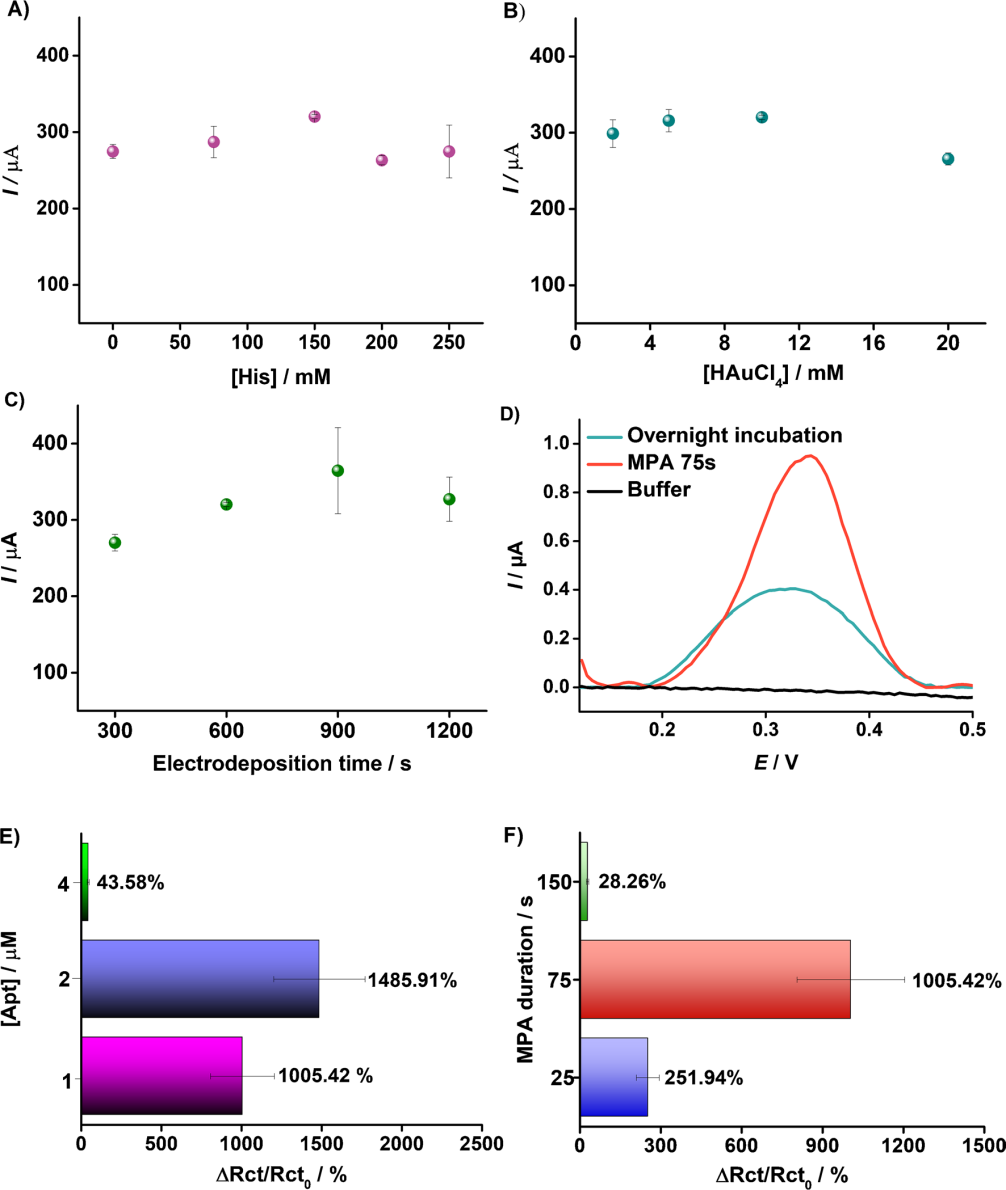
Optimization of Au electrodeposition process: the influence of histidine concentration (**A**), HAuCl_4_ solution concentration (**B**), and electrodeposition time on the peak anodic current during CV measurements in 5 mM [Fe(CN)_6_]^3−/4−^ in 0.1 M KCl (**C**). Optimization of Apt immobilization procedure: immobilization procedure (MPA vs ON) by recording the DPV signal of the Apt Fc label in 0.1 M KCl (**D**), Apt concentration (1, 2, and 4 µM) (**E**), and duration of MPA (25, 75, and 150 s) (**F**), EIS measurements performed in 5 mM [Fe(CN)_6_]^3−/4−^ in 0.1 M KCl

The resulting C-PE/AuNSs platforms were analyzed electrochemically by CV measurements in 5 mM [Fe(CN)_6_]^3−/4−^ in 0.1 M KCl. The parameter values and their influence on the anodic current peak are shown in Fig. 4A-C. As shown, using 150 mM of histidine in a 10 mM HAuCl_4_ solution and an electrodeposition period of 600 s led to the most favorable and reproducible platform, with the highest charge transfer of [Fe(CN)₆]^3^⁻/^4^⁻ redox probe. Thus, the cauliflower-like nanostructure-based platform was chosen to be optimal and was further used for the development of the VAN aptasensor.

### Aptamer immobilization optimization

In the aptasensor development process, the surface of the electrode was modified with the VAN-specific aptamer sequence, for the specific recognition and target binding. To graft the aptamer on the C-PE/AuNSs platform, two different deposition methods were investigated using a fixed Apt concentration (2 µM): overnight incubation (ON) and multipulse amperometry (MPA). The grafting efficiency was evaluated by measuring in 0.1 M KCl using DPV the electrochemical signal of the Fc group grafted onto the Apt. As can be observed in Fig. 4D, when immobilizing the Apt through MPA, a 238% higher signal of the Fc label could be detected than the signal attained after ON (0.95 µA vs 0.40 µA). Thus, using MPA, the next optimization parameter was the Apt concentration, where three different concentrations were questioned (1, 2, and 4 µM). Initially, the EIS analytical response (calculated as the percentage of resistance to electron transfer increase after Apt immobilization; or ΔRct/Rct_0_ (%)) showed an upward trend as the concentration of Apt increased from 1 to 2 µM (from 1005.42 to 1485.91%). However, beyond this optimal concentration, higher concentrations resulted in a decrease in the measured signal (43.59%), by inducing an overly dense coverage and hindering both target interaction and electron transfer processes (Fig. 4E). Finally, the effect of the immobilization time by MPA was tested (25 s, 75 s, and 150 s), where it could be observed that the EIS analytical response increased with the increase of the immobilization duration, reaching a maximum at 75 s (Fig. 4F). Longer durations caused the removal of the Apt and damage to the C-PE/AuNSs; a duration time of 75s was considered optimal and used for Apt immobilization by MPA.

### Electrochemical characterization of the aptasensor

The VAN-specific Apt, modified with a terminal thiol at the 5′ end and an Fc redox label at 3′ end was immobilized onto the AuNSs-enriched C-PE platform via thiol-gold chemistry.

The Apt sequence chosen for this study was previously designed during the SELEX procedure [24] to have a structure-switching functionality, meaning upon target binding it undergoes through a conformational change, which alters the distance between the redox label and the electrode surface and changes the measured current intensity in a proportional manner to the analyte concentration. Our platform lacked the sensitivity and the reproducibility for direct assessment via the Fc redox label. When measuring the Fc signal in 0.1 M KCl solution via DPV and CV, the recorded current intensity values before and after VAN incubation were very low (under 1 µA) and irreproducible. Therefore, for VAN detection and quantification, EIS spectra before and after incubation with VAN were used, with an external redox probe ([Fe(CN)_6_]^3−/4−^), free in solution.

After immobilization of Apt through the previously optimized MPA method, the surface of the electrode was blocked with a short alkanethiol through the backfilling method to mitigate nonspecific adsorption on the electrode surface. As a blocking agent, a 100 µM MCH solution in TRIS buffer was used, with the same MPA immobilization procedure, as opted for the Apt immobilization step.

**Fig. 5:**
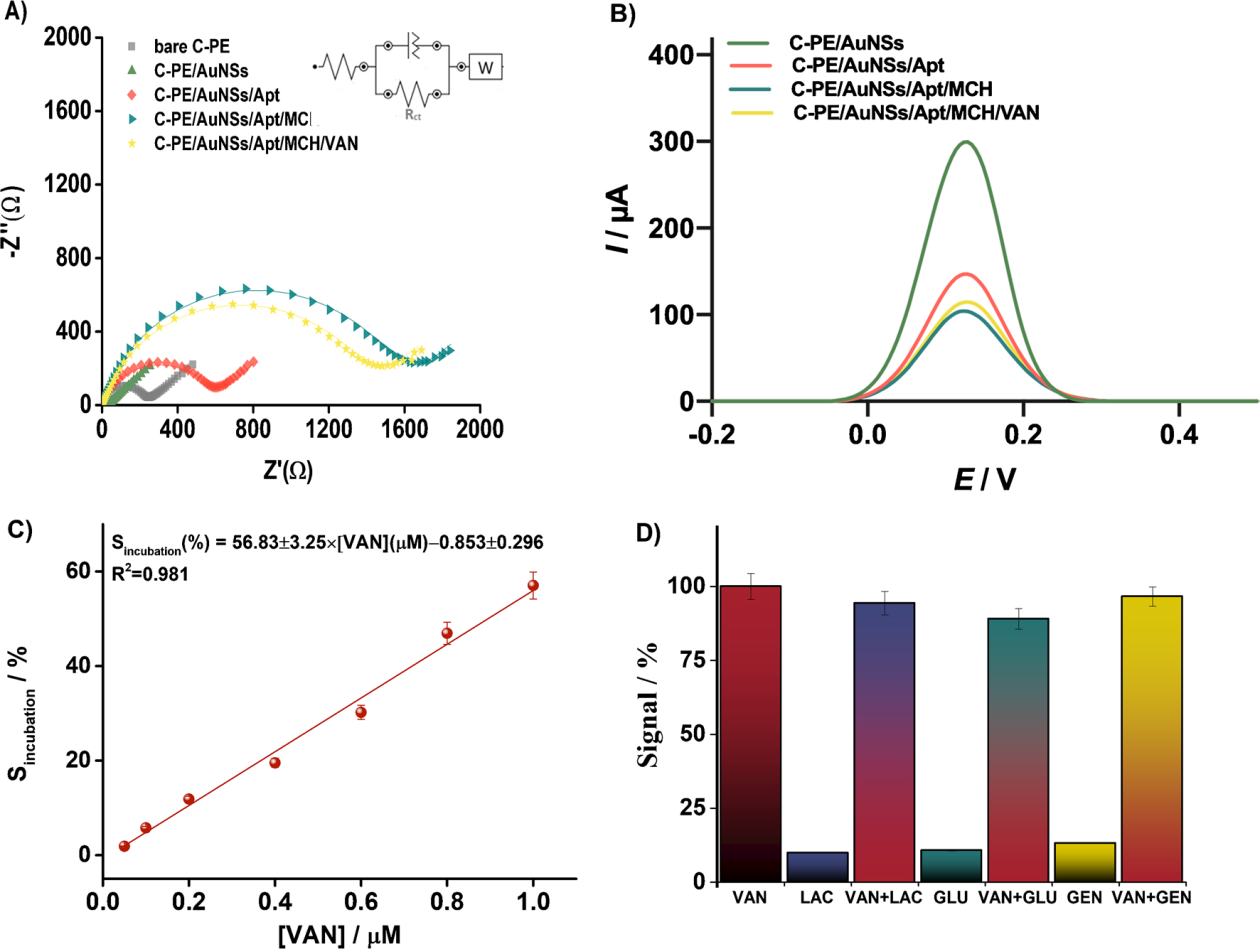
Electrochemical characterization of each step in the aptasensor development process and interaction with 0.2 µM VAN; analysis in 5 mM [Fe(CN)_6_]^3−/4−^ redox probe in 0.1 M KCl: Nyquist plots (60 frequencies, 1 MHz – 0.1 Hz at OCP; the experimental points are represented through symbols and the fitting through the continuous line; inset: the equivalent circuit used for fitting) (**A**); DPVs (− 0.1 V → + 0.4 V, 50 mV amplitude, 10 mV s^–1^) (**B**); EIS responses (S_incubation_ (%)) obtained in 5 mM [Fe(CN)_6_]^3−/4−^ after incubation of the aptasensor with different concentrations of VAN (0.05, 0.1, 0.2, 0.4, 0.6, 0.8, and 1 µM) (**C**); signal responses (%) obtained after the analysis of 1 µM interferent alone and equimolar (1 µM each) mixtures of interferent and VAN out of 1 µM VAN response (**D**). Error bars represent the standard deviation of at least 3 measurements

The stepwise fabrication of the electrochemical sensor was characterized by electrochemical methods, such as EIS (Fig. 5A) and DPV (Fig. 5B), using a 5 mM [Fe(CN)_6_]^3−/4−^ solution in 0.1 M KCl. The results obtained by the two techniques (EIS and DPV) were coherent, confirming the phenomena occurring after each step at the surface of the electrode.

By analyzing the Nyquist plots obtained after EIS characterization (Fig. 5A), a decrease in the electron transfer resistance (R_ct_) was observed by decorating the working electrode of C-PE with cauliflower-like AuNSs (from 235 to 36.6 Ω), thereby increasing the active surface area and improving the sensitivity by the enhanced current density of the electrochemical signal. As expected, R_ct_ increased after Apt immobilization (from 36.6 to 585.6 Ω) and MCH blocking steps (from 585.6 to 1540 Ω), which is attributed to the Apt/organic film-modified surface, which hinders electron transfer at the electrode platform/solution interface. Finally, after incubation with 0.2 µM VAN, a decrease in Rct was observed (from 1540 to 1380 Ω), which can be attributed to two main phenomena: (i) the conformational changes that occur in Apt’s secondary structure during target interaction and complex formation, thus enhancing the electron transfer at the platform’s surface; (ii) the positively charged nature of VAN at physiological pH, which could mediate the electrostatic attraction of the negatively charged [Fe(CN)_6_]^3−/4−^.

Figure 5B shows DPVs performed in 5 mM solution of [Fe(CN)_6_]^3−/4−^ in 0.1 M KCl at C-PE/AuNSs, C-PE/AuNS/Apt, C-PE/AuNS/Apt/MCH, and after binding with 5 µM VAN. After the immobilization of the aptamer (Fig. 5B, red curve), a substantial decrease in the DPV peak current was observed compared to the characteristic behavior of [Fe(CN)_6_]^4−/3−^ redox probe at C-PE/AuNS (Fig. 5B, green curve). This change is attributed to the negative charge of the DNA aptamer, which repels the negatively charged [Fe(CN)_6_]^4−/3−^ anions, hindering electron transfer between the redox probe and the electrode surface. A further decrease in peak current was observed after blocking the electrode with MCH (Fig. 5B, blue curve), indicating a further decrease in the electron-transfer rate due to the filling of empty spaces on the C-PE/AuNS surface. After incubating the aptasensor with VAN, the DPV peak current increased (Fig. 5B, yellow curve). This result suggests a conformational change within the aptamer upon binding with VAN, thereby enhancing the accessibility of the [Fe(CN)_6_]^3−/4−^ redox couple to the sensor surface.

### Analytical performance of the proposed aptasensor

The analytical performance of the developed aptasensor was evaluated in the optimized conditions against various concentrations of VAN by measuring the decrease in the *R*_*ct*_ value in EIS (S_incubation_ expressed as *ΔR*_*ct*_*/R*_*ct0*_ (%), where *ΔR*_*ct*_ = *R*_*ctMCH*_*—R*_*ctVAN*_ and *R*_*ct0*_ = *R*_*ctMCH*_) after target incubation for 45 min. The outcome indicated a linear correlation between S_incubation_ (%) and the incubated concentration of VAN within the range of 5.0 × 10^−8^ to 1 × 10^−6^ M, with the following equation: S_incubation_ (%) = 56.83 ± 3.25 × [VAN] (µM) – 0.853 ± 0.296 (*R*^2^ = 0.981 (Fig. 5C). The detection limit was determined using MacDougall et al. method [34] (St-Sb ≥ 3σ) and it was found to be 1.721 × 10^−9^ M. Using the same method (St-Sb ≥ 10σ), the limit of quantification (LOQ) was calculated to be 5.216 × 10^−9^ M. St represents the gross analytical signal, and Sb represents the measured signal for the average blank and σ the standard deviation of the calibration curve.

To assess the analytical performance of the developed aptasensor, a comparison with some of the latest electrochemical detection methods for VAN in different real samples was summarized. As shown in Table S1, the great advantage of the aptasensor developed in this study compared to the previous aptasensor is its user-friendliness, highlighted by the simplified fabrication steps and analysis time (47 min), very good LR and LOD and, most importantly, flexibility and portability, which can enable in situ or point-of-care testing. When comparing different biorecognition elements used in sensor fabrication, both Apts and MIPs enhance the analytical performance of the sensor, bringing advantages in sensitivity, specificity, stability, cost, and time. The advantages of Apts come from the higher specificity tailored through SELEX, and the versatility of the different functional groups grafted, much more suitable when questioning various nanomaterial-enriched sensing platforms.

### Interference studies

VAN is frequently used in combination with a second antibiotic, typically gentamicin (GEN), for the treatment of severe infections caused by MRSA [35]. The selectivity of the developed aptasensor was investigated towards GEN and two other biomolecules frequently found at high levels in the selected real samples (milk and human serum), lactose (LAC), and glucose (GLU).

As shown in Fig. 5D, the EIS responses when incubating the interferents alone (LAC, GLU, and GEN) did not produce significant variations (9.78%, 10.65%, and 13.03% of the VAN’s signal for 1 µM concentration), whereas when incubating the equimolar mixture, the obtained signal change was similar to that caused by VAN alone (94.37%, 89.05%, and 96.58%, respectively), thus highlighting the selectivity of the aptasensor towards VAN.

### Real samples analysis

To verify the performance and accuracy of the developed sensor, a known quantity of VAN was added to different real samples, and the recovery rates using the calibration curve were determined. Milk samples with different fat contents (0.1%, 1.5%, and 3%) and artificial human serum were chosen as real samples based on the prospective applicability of the aptasensor in food safety and therapeutic drug monitoring. After a 1:10 dilution and incubation with the spiked real samples, the EIS responses were assessed. The results are presented in Table 1. Each measurement was conducted in triplicate. The recovery values ranged from 96.2 to 104.4%, with a good RSD (< 4%), suggesting that this method can be reliably used for real sample analysis. The observed differences in recovery and precision across the various matrices (milk at different fat concentrations and artificial human serum) highlight the influence of the matrix on the performance of the sensor. Matrix components, such as proteins, lipids, and other substances present in milk and serum, can interact with the target analyte or sensor surface, affecting the binding efficiency and the signal generated. For example, in milk with 0.1% and 1.5% fat content, the recovery rates were close to 100% (99.10% and 98.10%, respectively), suggesting that matrix effects were minimal in these cases. However, in milk with 3%, the recovery was slightly higher at 104.40%, which could be due to the presence of higher concentrations of components that may have affected the binding interaction or the sensor sensitivity. In the case of artificial human serum, the recovery was lower (96.20%), and the RSD increased to 3.68%, indicating that serum proteins or other factors might interfere more significantly with the analyte detection, leading to slightly lower accuracy and higher variability in the measurements.

**Table 1:** VAN recovery rates obtained from spiked milk and serum samples by EIS

Real sample	Added (µM)	Found (µM)	Recovery (%)	RSD (%)
Milk 0.1%	1.00	0.99	99.10	0.08
Milk 1.5%	1.00	0.98	98.10	2.32
Milk 3%	1.00	1.04	104.40	2.56
Artificial human serum	1.00	0.96	96.20	3.68

## Conclusions

In this work, a flexible and portable electrochemical aptasensor was developed for the in situ detection of VAN from biological and food samples to assure maximum efficacy and minimum toxicity of the VAN treatment.

The electrochemical aptasensor was based on carbon electrodes (C-PE) lab-printed on a flexible substrate and modified with cauliflower-shaped AuNSs, for increased electrochemical performance and active surface for VAN-specific aptamer immobilization as a biorecognition element. The VAN-aptamer affinity was fully characterized by ITC and SPR. Different readout methods were tested (Fc label vs redox probe in solution), with the label-free detection through EIS being the most sensitive, allowing the VAN detection over a wide concentration range and LOD in the nanomolar range.

The developed aptasensor showed good selectivity and it was successfully applied for the analysis of real samples of spiked milk and human serum. The flexible, portable developed aptasensor, together with portable potentiostats equipped with the EIS technique, could be integrated into handheld, user-friendly devices, making it more accessible for real-world applications such as on-field analysis and point-of-care monitoring of VAN.

## Supplementary Information

Below is the link to the electronic supplementary material.

Supplementary file1 (DOCX 322 KB)

## Data Availability

No datasets were generated or analysed during the current study.
