# Supplementary material for: Electrochemical aptasensor for the selective detection of vancomycin based on nanostructured “in-lab” printed electrodes
Source: Mikrochim Acta. 2025 Jan 25;192(2):107. doi: 10.1007/s00604-025-06952-1 (PMC11762413; doi:10.1007/s00604-025-06952-1)
Supplement: Supplementary file 1 — Supplementary file1 (DOCX 322 KB) [file 604_2025_6952_MOESM1_ESM.docx]

***Supplementary Material***

**Electrochemical Aptasensor for the Selective Detection of Vancomycin Based on Nanostructured “In-lab” Printed Electrodes**

**Malek Bibani^1,2#^(**[0000-0002-0950-0875](https://orcid.org/0000-0002-0950-0875)**), Magdolna Casian^1#^(**[0000-0002-0070-4142](https://orcid.org/0000-0002-0070-4142)**), Bogdan Feier^1^(**[0000-0002-9309-0951](https://orcid.org/0000-0002-9309-0951)**), Diana Bogdan^3^ (**[0000-0002-5046-4837](https://orcid.org/0000-0002-5046-4837)**), Oana Hosu-Stancioiu^1^(**[0000-0002-3579-5195](https://orcid.org/0000-0002-3579-5195)**), Nadia Ktari^2^ (**[0000-0001-9823-9818](https://orcid.org/0000-0001-9823-9818)**), Rafik Kalfat^2^ (**[0000-0001-6638-9133](https://orcid.org/0000-0001-6638-9133)**), Cecilia Cristea^1^ (**[0000-0002-4158-3324](https://orcid.org/0000-0002-4158-3324)**)**

*^1^Department of Analytical Chemistry, Faculty of Pharmacy, “Iuliu Hațieganu” University of Medicine and Pharmacy, 4 Pasteur Street, 400349, Cluj-Napoca, Romania*

*^2^Laboratoire Matériaux, Traitement et Analyse, INRAP, BiotechPole Sidi-Thabet, 2020 Ariana, Tunisia*

*^3^National Institute for Research and Development of Isotopic and Molecular Technologies, 67-103 Donat St., 400293 Cluj-Napoca, Romania*

*# equal contribution*

[feier.george@umfcluj.ro](mailto:feier.george@umfcluj.ro)

**
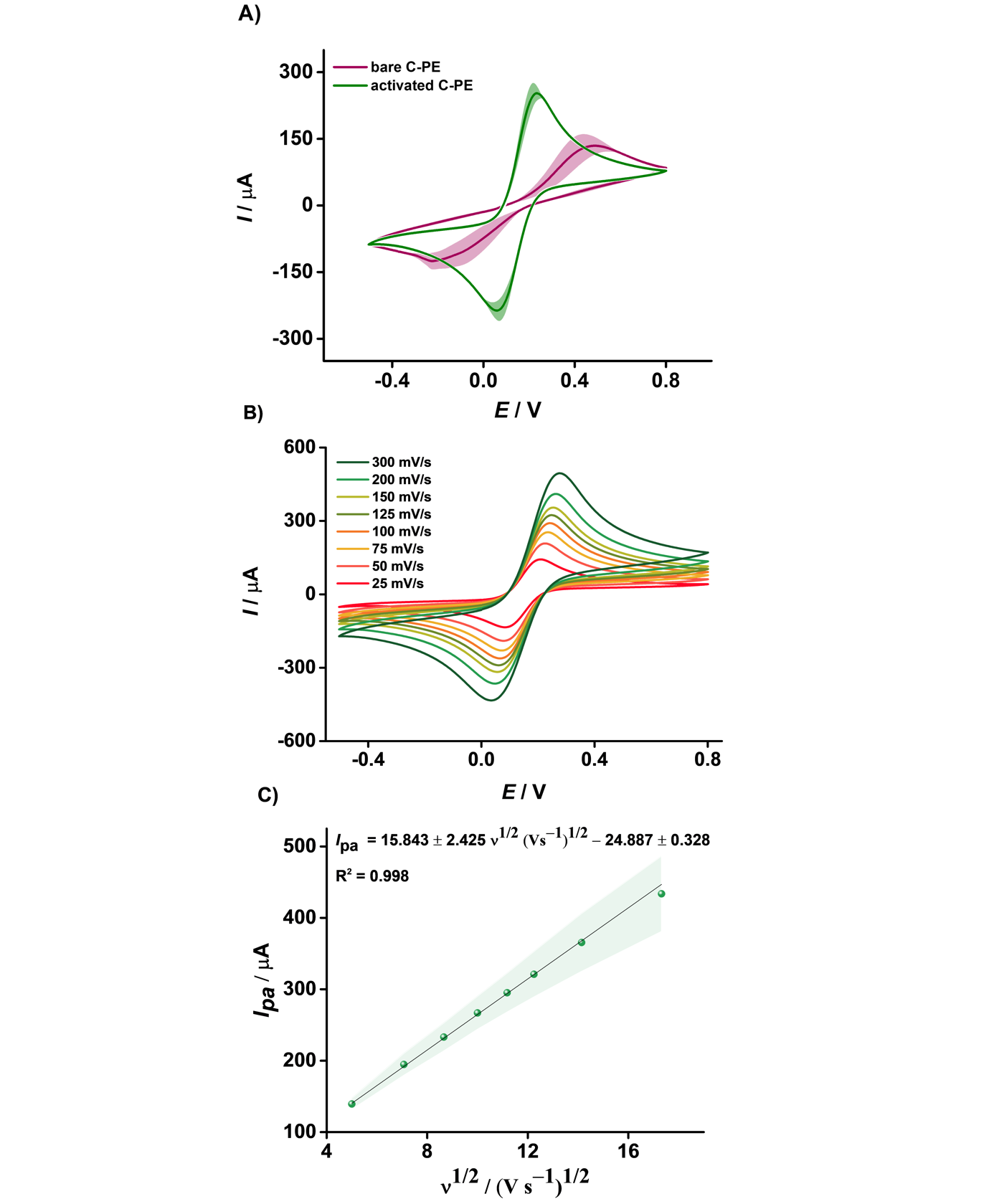
**

**Fig. S1. A)** The influence of C-PE surface activation by chronoamperometry with 1 M Na_2_CO_3_ on CV voltammogram of 5 mM [Fe(CN)_6_]^3−/4−^ in 0.1 M KCl; **B)** CVs at scan rates between 25 and 300 mV/s for bare C-PEs and **C)** the corresponding plot of anodic peak current versus square root of scan rate. The CV measurements were performed in 5 mM [Fe(CN)_6_]^3−/4−^ in 0.1 M KCl. The shaded area represents the standard deviation of at least 3 measurements.

**Table S1.** Analytical performance of different electrochemical sensors for VAN detection

| (Bio)recognition element | Sensor configuration | Electrochemical technique | Analytical performances | | | Fabrication + analysis time | Real sample | Ref. |
| --- | --- | --- | --- | --- | --- | --- | --- | --- |
|  |  |  | **LR (nM)** | **LOD (nM)** | **Interferences** |  |  |  |
| MIP | GCE-MIP/alginate@TiO_2_ NPs | DPV | 0.01 – 0.1 | 0.003 | DOP, AA, PAR, Na^+^, SO4^2–^, K^+^, Cl^–^ | ~45 min | Artificial serum and tap water | [1] |
| MIP-PEP | GCE/AuNPs/MIP-PEP | EIS | 0.01 –10^5^ | 0.01 | TET, CTC, OFL, BPA, STR, RIF | ~14,5 h | Fetal calf serum, probiotic drink and honey | [2] |
| - | SPCE/ AuNSs | DPV | 10^3^ – 10^5^ | 290 | AMX, PenV, PenG, OXA, CFX, GEN, PAR, AA | ~40 min | Artificial human serum | [3] |
| MOF-PAA | GCE/MOF-PAA | CV | 1 – 100 | 1 | CIP, GEN | >24 h | Human urine and serum | [4] |
| Apt | Wire electrodes/Apt | SWV | Calibration free sensor  for continuous monitoring | | | | In vivo/ Rat plasma | [5] |
| Apt | C-PE/ AuNSs/ Apt/MCH | EIS | 50 – 10^3^ | 1.721 | GEN, LAC, GLU | ~45 min | Human artificial serum and milk | This work |
| *AA – Ascorbic acid; AMX – Amoxicillin; Apt – Aptamer; AuNPs – Gold nanoparticles; AuNSs – Gold nanostructures; BPA – Bisphenol A; CFX – Cefalexin; CIP – Ciprofloxacin; C-PE – Carbon printed electrode; CTC – Chlorotetracycline; DOP – Dopamine; GCE – Glassy carbon electrodes; GEN – Gentamycin; GLU – Glucose; LAC – Lactose; MIP – Molecularly imprinted polymer; MOF – Metal organic framework; OFL – Ofloxacin; OXA – Oxacilin; PAA – Poly(acrylic acid); PAR – Paracetamol; PenG – Penicillin G; PenV – Penicillin V; PEP – Peptide; RIF – Rifampicin; SPCE – Screen printed carbon electrodes; STR – Streptomycin; TET – Tetracycline; TiO_2_ – Titanium dioxide; DPV – Differential pulse voltammetry; EIS – Electrochemical impedance spectroscopy; C – Cyclic voltammetry; SWV – Square wave voltammetry.* | | | | | | | | |

**References**

1. Cetinkaya A, Yıldız E, Kaya SI, Çorman ME, Uzun L, Ozkan SA. A green synthesis route to develop molecularly imprinted electrochemical sensor for selective detection of vancomycin from aqueous and serum samples. Green Analytical Chemistry. 2022 Aug 1;2:100017.

2. Tan F, Zhai M, Meng X, Wang Y, Zhao H, Wang X. Hybrid peptide-molecularly imprinted polymer interface for electrochemical detection of vancomycin in complex matrices. Biosens Bioelectron. 2021 Jul 15;184:113220.

3. Blidar A, Feier B, Pusta A, Drăgan AM, Cristea C. Graphene–Gold Nanostructures Hybrid Composites Screen-Printed Electrode for the Sensitive Electrochemical Detection of Vancomycin. Coatings 2019, Vol 9, Page 652. 2019 Oct 10;9(10):652.

4. Gill AAS, Singh S, Agrawal N, Nate Z, Chiwunze TE, Thapliyal NB, et al. A poly(acrylic acid)-modified copper-organic framework for electrochemical determination of vancomycin. Microchimica Acta. 2020 Jan 1;187(1):1–9.

5. Dauphin-Ducharme P, Yang K, Arroyo-Currás N, Ploense KL, Zhang Y, Gerson J, et al. Electrochemical Aptamer-Based Sensors for Improved Therapeutic Drug Monitoring and High-Precision, Feedback-Controlled Drug Delivery. ACS Sens. 2019 Oct 10;4(10):2832.
